# Climate change, its impact on emerging infectious diseases and new technologies to combat the challenge

**DOI:** 10.1080/22221751.2024.2356143

**Published:** 2024-05-20

**Authors:** Hongyan Liao, Christopher J. Lyon, Binwu Ying, Tony Hu

**Affiliations:** a Department of Laboratory Medicine, West China Hospital, Sichuan University, Chengdu, People’s Republic of China; b Center for Cellular and Molecular Diagnostics and Department of Biochemistry and Molecular Biology, Tulane University School of Medicine, New Orleans, LA, United States

**Keywords:** Emerging infectious disease, climate change, transmission, outbreak, early diagnosis

## Abstract

Improved sanitation, increased access to health care, and advances in preventive and clinical medicine have reduced the mortality and morbidity rates of several infectious diseases. However, recent outbreaks of several emerging infectious diseases (EIDs) have caused substantial mortality and morbidity, and the frequency of these outbreaks is likely to increase due to pathogen, environmental, and population effects driven by climate change. Extreme or persistent changes in temperature, precipitation, humidity, and air pollution associated with climate change can, for example, expand the size of EID reservoirs, increase host–pathogen and cross-species host contacts to promote transmission or spillover events, and degrade the overall health of susceptible host populations leading to new EID outbreaks. It is therefore vital to establish global strategies to track and model potential responses of candidate EIDs to project their future behaviour and guide research efforts on early detection and diagnosis technologies and vaccine development efforts for these targets. Multi-disciplinary collaborations are demanding to develop effective inter-continental surveillance and modelling platforms that employ artificial intelligence to mitigate climate change effects on EID outbreaks. In this review, we discuss how climate change has increased the risk of EIDs and describe novel approaches to improve surveillance of emerging pathogens that pose the risk for EID outbreaks, new and existing measures that could be used to contain or reduce the risk of future EID outbreaks, and new methods to improve EID tracking during further outbreaks to limit disease transmission.

## Introduction

### Emerging infectious disease

**Figure 1.**
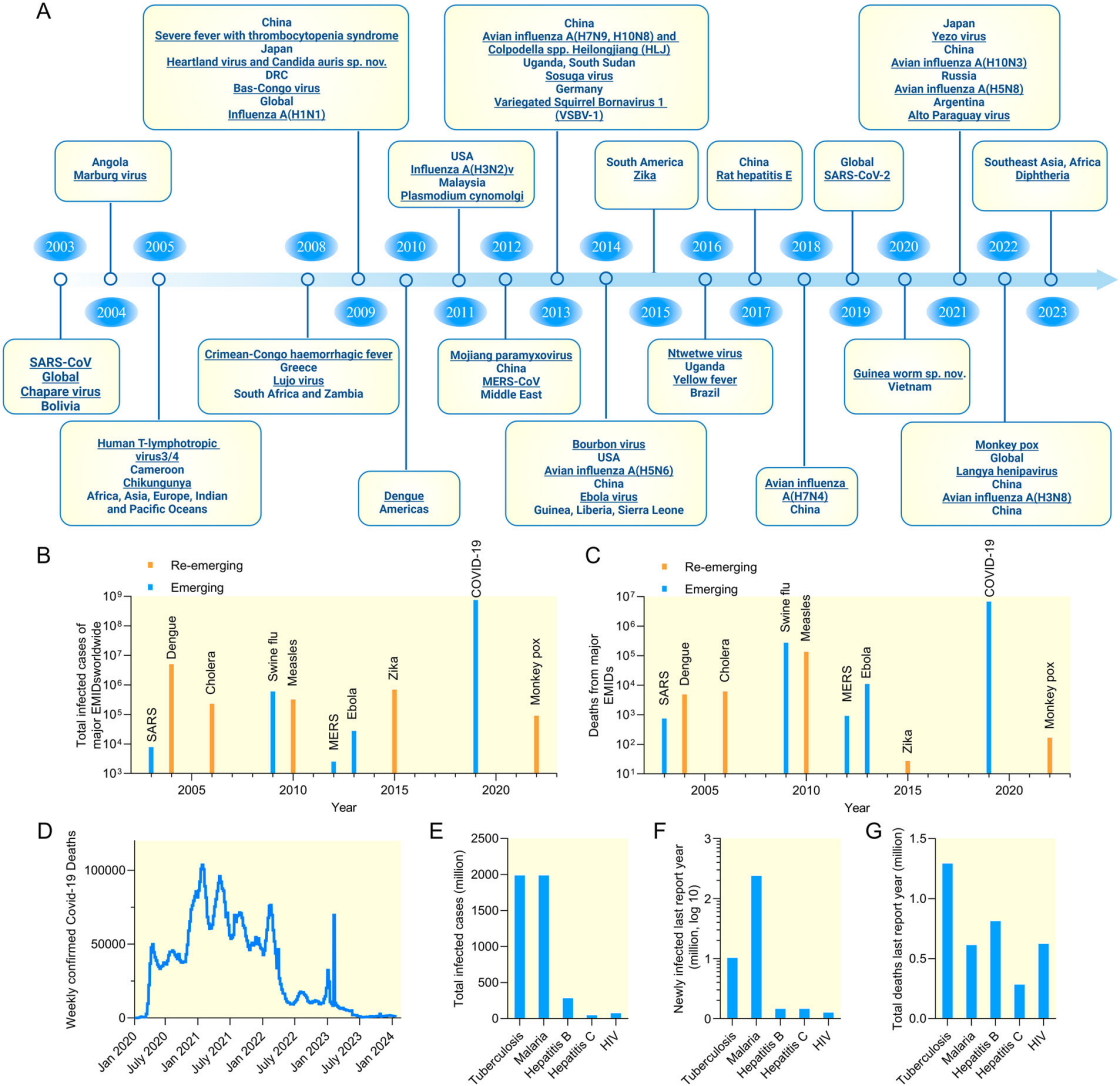
Major EID outbreaks in the past 20 years demonstrating *de novo* jumps into human populations or increased geographic range or infectivity: A, timeline of emerging or re-emerging epidemic or endemic microbial infections. B, total estimated infected cases and C, deaths from major EID epidemics between 2003 and 2023. D, weekly confirmed deaths refer to the cumulative number of confirmed deaths over the previous week of Covid-19 since Jan 2020. E-G, leading EIDs with estimated total infected cases (E), newly infected cases (F) and deaths (G) in the latest report year (tuberculosis, 2022; malaria, 2021; hepatitis B, 2019; hepatitis C, 2019; HIV, 2022)(data sources GOV.UK, World Health Organization, US Centers for Disease Control and Prevention, European Centre for Disease Control and Prevention and OurWorldindata). Figure created with Biorender.com and Graphpad 9.4.1.

Emerging infectious disease (EID) outbreaks are defined by a cluster of cases caused by emerging pathogens previously unknown to cause disease or re-emerging pathogens, which have previously caused disease but now reveal increased infectivity or extended transmission ranges that improve their ability to cause disease in human populations [1]. The single largest EID outbreak since the development of modern medicine, the 1918 influenza pandemic, was responsible for approximately 500 million infections and 50 million deaths [2]. However, population growth and mobility, climate change, pollution and environmental degradation effects, and the aging of the global population have increased the risk of future pandemics. Multiple EID outbreaks have been observed in the past decades, but these have primarily failed to establish endemic infections (with the exception of SARS-CoV-2). Most pathogens that have made this transition started as spillover events from animal reservoirs and sufficiently adapted to their new human hosts to allow direct host-to-host transmission through body fluid exchange or respiratory droplet exposure, including HIV-1 and SAR-CoV-2. Neither of the EID pandemics caused by these pathogens produced rapid mortality rates to rival those observed in the 1918 influenza pandemic due to disease-specific differences as well as advances in disease diagnosis, tracking, and prevention approaches. Nonetheless, both continue to have ongoing effects on global health, as there have been >772 million confirmed SAR-CoV-2 infections and >6.98 million deaths worldwide [3] and >85.6 million HIV-1 infections and >40.4 million deaths [4] since their respective outbreaks. Several recent EID outbreaks – including two caused by respiratory viruses closely related to SAR-CoV-2 (SAR-CoV and MERS-CoV, likely originated from bats and were directly transmitted to humans from market civets and camels, respectively) – did not succeed in establishing persistent human reservoirs, but the growing frequency of such spillover EID outbreaks highlights the importance of zoonotic sources and growing risk for new epidemics or pandemics (Figure 1). Multiple recent EID outbreaks (e.g. the 2003 SARS and the 2012 MERS outbreaks, the 2009 swine flu pandemic, the 2013–2016 Ebolavirus epidemic, the 2014 chikungunya outbreak in the Western Hemisphere and the 2015 Zika virus epidemic) have exhibited significant morbidity and mortality and large geographic ranges, however, indicating the potential for even more severe outcomes in future outbreaks. Several additional pathogens are also considered capable of producing EID outbreaks, including *Escherichia coli* O157:H7, hantavirus, dengue virus and West Nile virus, while malaria, tuberculosis (TB), cholera, and influenza are often considered reemerging diseases with the potential for new outbreaks. New rapid point-of-care (POC) diagnostics and vaccine development methods may attenuate the spread of future EID outbreaks if applied in conjunction with effective disease prevention and control methods. However, EIDs remain a major health concern and can still cause high mortality despite these new capabilities and increased access to improved sanitation, hygiene and medical care [5], due in part to increased exposure of naïve populations to EID pathogens due to population expansions and migrations associated with climate change.

### Climate change-a threat to public health

Climate change is unequivocally acknowledged as an urgent global public health menace by the World Health Organization (WHO), as profound and enduring shifts in temperature, precipitation, humidity, and air and water quality are already exacting a devastating toll on human health, amplifying morbidity and mortality rates worldwide [6]. Climate change can enhance transmission of food-, water- and vector-borne diseases (FBDs, WBDs and VBDs) by increasing the area in which or length of time when conditions remain favourable for outbreaks and transmission of emerging or reemerging infectious disease through effects on pathogen or host health, replication, and dissemination or migration [7]. One recent modelling study of 3000 mammalian species found that their predicted migration patterns could lead to more than 4000 instances of cross-species viral transmission in the next 50 years if the world warms by 2°C, and thus climate change could easily become the dominant anthropogenic force in viral cross-species transmission [8]. In fact, climate change-induced mortality and morbidity associated with infectious diseases are expected to rise globally [9]. The impact of climate change on fungal infections has been reviewed elsewhere [10], which also emphasizes the association of climatic factors and fungi-associated outbreaks. Here, we review the mechanisms of climate change altering the landscape of EIDs. At a global glance, we propose measures that have or could be taken to prevent or limit initial and subsequent transmission events. We also discuss emerging technologies that can be applied to generate more effective surveillance systems for early control of EID outbreaks.

## Effects of climate change on EID transmission

**Figure 2.**
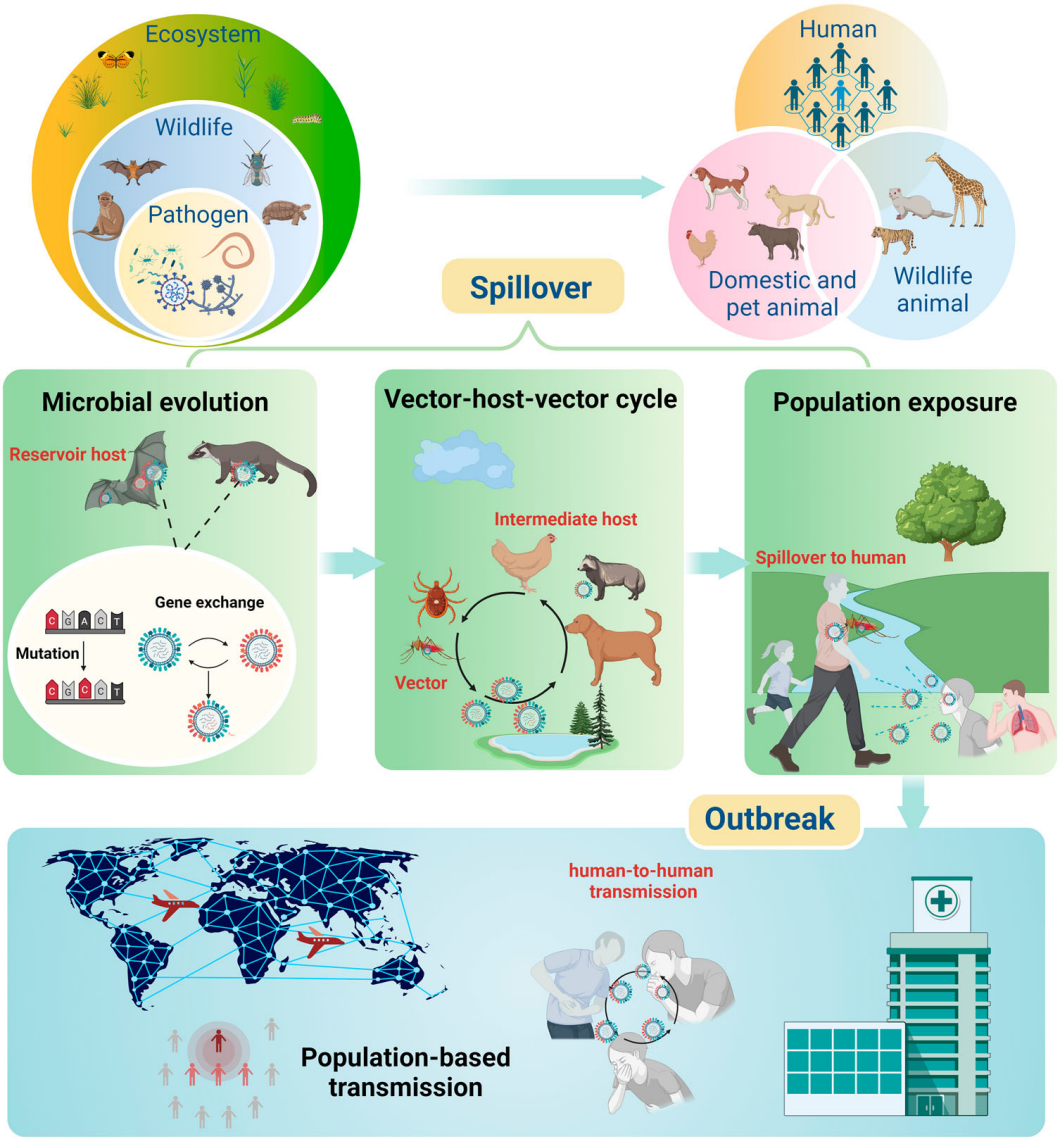
The processes of pathogen spillover and EID outbreak: pathogens, including bacteria, virus, fungi, parasite and protozoan from wild animals move into domestic, pets, wild animals or directly to humans. This spillover process is further detailed in steps including long-term evolution of the pathogen itself to gain the ability to jump from the original reservoir host to intermediate host, formation of cyclic vector-host-vector transmission cycle and the population-based transmission after initial exposure event, and expansion of the pathogen’s geographical range beyond the region of spillover enabled by human-to-human transmission that leads to the EID outbreak. Figure created with Biorender.com.

A diverse array of factors can influence EID spillover and outbreak, including new mutations and viral recombination and reassortment events that alter the species-specificity of a pathogen, changes that affect the geographic ranges of environmental pathogens or animal vectors, and human migration and habit destruction events that can lead to new geographic overlaps between a human population and a pathogen or its vector(s) [11]. Repeated direct or indirect contact between a human population and a pathogen or a pathogen reservoir are usually required to permit sufficient cross-species transmission and mutation and selection events to allow a pathogen to evolve a capacity for efficient human-to-human transmission [12]. Spillover events leading to population-based transmission in humans can occur through periodic or continuous exposure to reservoirs of pathogens or via cyclic vector-host-vector transmission routes. Environmental encroachment events, due to climate change or other anthropogenic causes, and the inherent mutation rates of pathogens present in the affected regions create fertile opportunities for infectious agents to adapt to new ecological niches, interact with and adapt to new hosts through repeat exposure to promote zoonotic infection, host shift, and population-based transmission (Figure 2) [11].

**Figure 3.**
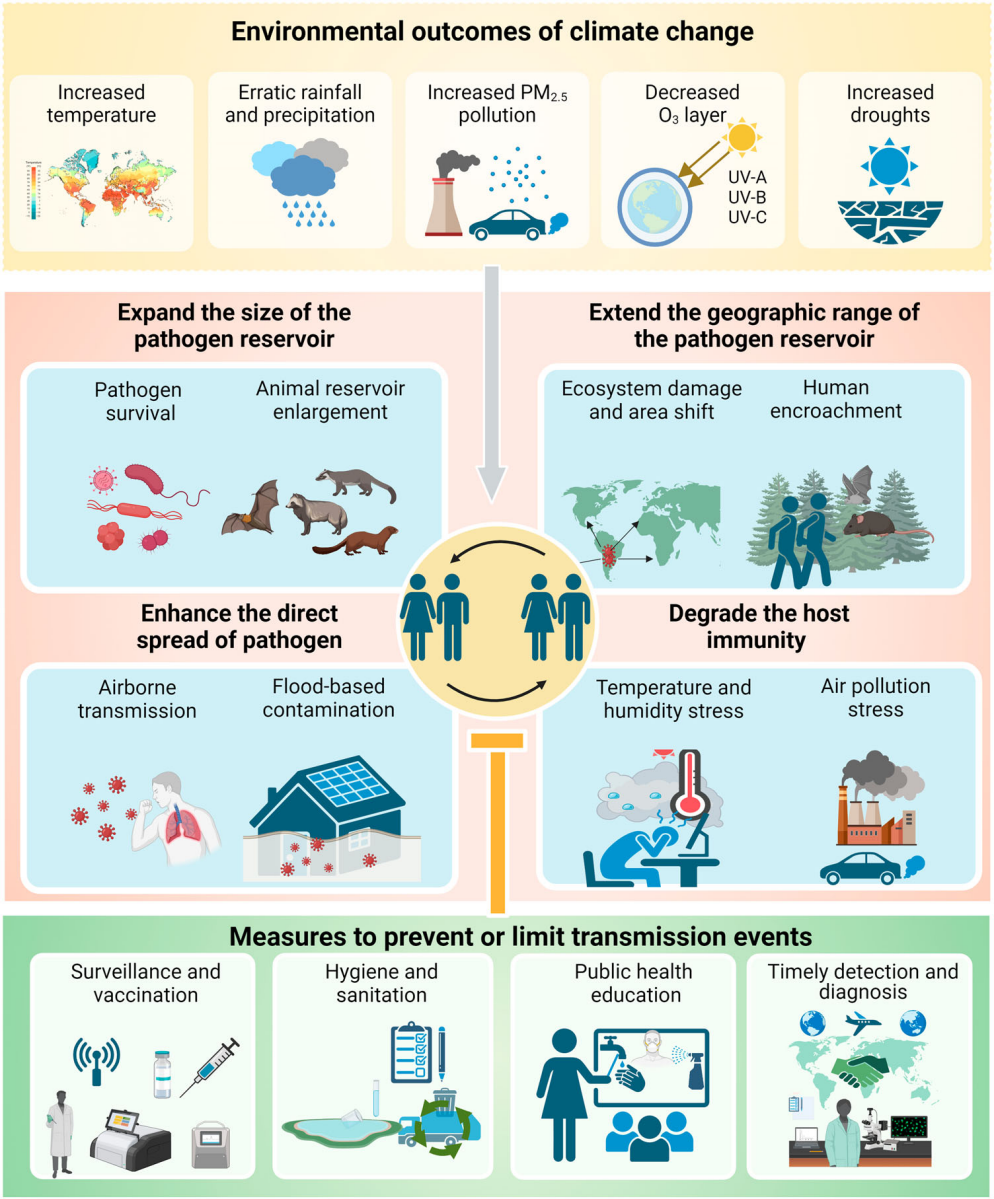
Environmental outcomes of climate change, potential climate-regulated mechanisms effects that can promote EID outbreaks, and measures that can be taken to prevent or limit EID transmission events. Figure created with Biorender.com.

Environmental modifications associated with climate change can expand the size of a pathogen reservoir or its host population(s), alter or increase their geographic range through ecosystem damage, increase the likelihood of EID spillover events, and decrease the health of host populations to enhance the risk of successful spillover events and outbreaks (Figure 3). Climate change can increase pathogen abundance by enhancing the survival and reproduction of pathogens or their hosts in existing environmental niches or expanding the number, size, or reproductive potential of these niches in an existing or expanded geographic range. Changes in temperature, precipitation, and humidity can alter the size and distribution of pathogen and host populations to influence pathogen and host development, replication and survival [13].

Respiratory virus transmission may provide the best documented evidence for temperature- and humidity-mediated effects on pathogen transmission in human populations, since endemic influenza A, respiratory syncytial virus (RSV) and coronaviruses all exhibit clear seasonal transmission peaks. This may be due to sunlight, UV, temperature and humidity that affect virus survival in aerosols and on surfaces, as well as behavioural changes with increased close contacts arising from individuals spending more time indoors during the winter season [14]. Interestingly, Mordecai EA *et al.* revealed that mosquito-borne pathogens have peak transmission at a temperature range of 23–29°C and decline to zero below 9–23°C and above 32–38°C. By analyzing the reproductive number (R_0_) of pathogens from the aspect of thermal biology, such findings provide direct evidence of how climate change could affect the transmission of ectothermic vectors and parasites [15]. In addition, extreme temperature increases can expand the risk of EID outbreaks in human populations, as demonstrated by the surge in SARS-CoV-2 cases observed worldwide during the summer of 2022, which appears to have been at least partially due to individuals congregating in climate controlled areas, such as shopping malls, particularly in regions where residential climate control was not widespread [16].

Climate change can have multiple effects to increase pathogen burden and disease transmission within animal reservoirs leading to disease spillover into human populations. For instance, variable seasonal extremes in temperature and rainfall associated with climate change can lead to rapid increases in the food supply of a disease reservoir, leading to a population explosion that cannot be controlled by its predators. Such changes may promote the risk for spillover events if they increase the geographic range or population density of a disease reservoir to promote *de novo* or existing direct or indirect interactions with susceptible human populations. The 1990s Hantavirus epidemic in the southwestern United States represents one such example, as it was linked to indirect human contact with a deer-mouse reservoir that experienced a 10-fold population density increase due to a short-term climate induced fluctuation that increased its food supply and suppressed its predator populations [17]. Similar long-term effects should also be possible if long-term climate change continues to favour increased interaction between a disease reservoir and a human population in a manner that is not adequately suppressed by a predator population.

Climate change can also extend or alter the geographic range of a pathogen reservoir via positive or negative effects on food supply, predator populations, and other factors that affect its current range and adjacent locales. For example, ecosystem damage may force a host population or its predators to range further to meet their basic environmental needs or to avoid increased competition or predation resulting from ecosystem changes. This may increase the number of infected hosts, their individual pathogen burden, and the chance for contact with a susceptible human or human-adjacent host population. Ecosystem damage can also have similar effects on human populations and domestic animals that may serve as gateway populations. In both cases, however, increased contact between a stressed host or susceptible host-candidate population may augment the risk for close contacts that lead to spillover events. For example, higher temperatures and meteorologic shifts associated with climate change can reduce surface water availability by decreasing the amount of rainfall or increasing the extent of evaporation, causing wildlife to frequent water source that they might otherwise avoid due to their frequent use by human or domestic animal populations, leading to direct or indirect contact between these groups [18]. However, such resource competition may not be necessary to promote spillover events. Research indicates that climate change has caused the Australian black flying fox population, a key reservoir of Hendra virus, to migrate 100 km south over the past 100 years, likely leading to observed spillover events in southern horse populations that led to human infections [19]. Climate change has also been linked to changes in the El Niño southern oscillation that have been linked to the geographic distribution of endemic cholera, in a manner that is not directly linked to increased rainfall and flooding [20].

EID species diversity increases as one proceeds from the poles to the equator [21], and environmental parameters that promote pathogen transmission at lower latitudes (e.g. increased temperature and precipitation) are hypothesized to drive this pattern [21]. A recent study modelling potential connections between climate variables and pathogen prevalence has also suggested that elevated temperatures and greater precipitation will accelerate the transmission of bird- and bat-associated bacteria or viruses to wild and domesticated animals and humans [22]. One example of the potential impact of this process was the recent COVID-19 pandemic, where a SARS-CoV-2 progenitor virus that probably originated in southeast Asian horseshoe bats (*Rhinolophus* sp.) is proposed to have spread to humans through an as-yet-unknown bridge host [23].

Changing climate conditions can affect EID transmission chains and routes by altering the geographic range and population sizes of potential pathogen and host species, leading to novel interactions between these species that can lead to establishment of a new pathogen reservoir and increase the risk for subsequent zoonotic disease transmission to a human population [8]. VBDs spread by blood-feeding arthropods such as mosquitoes, ticks, and fleas, are responsible for substantial morbidity and mortality as they transmit diseases (malaria, dengue, West Nile, and Lyme disease) that account for 22.8% of EID events worldwide [1]. These insect vectors are sensitive to environmental changes (rainfall, temperature and severe weather events) associated with climate change [24]. For example, temperature changes can affect the transmission period or the growth rate an insect vector (or its pathogen), by an analysis of mosquito-based transmission of dengue fever or *Plasmodium falciparum* malaria [25].

Drinking water safety is a key factor affecting EID transmission. Climate change can increase rainfall and flooding to contaminate water supplies of a human or human-adjacent host populations leading to an increased risk for outbreaks of WBDs caused by enteric bacteria and parasites such as *Salmonella* and *Cryptosporidium* [26]. In climate-vulnerable areas, for example, Bangladesh, drinking water safety could be threatened by climatic hazards, which leads to the prevalence of multiple WBDs and VBDs, such as cholera, dengue, and malaria [27].

Climate effects can also worsen air pollution, and both these factors can cause and synergically exacerbate detrimental effects on the host immune system. This can enhance respiratory disease transmission by affecting the lifetime of air-borne respiratory droplets and the pathogens they carry and via detrimental effects on the respiratory mucosa and immune system of at-risk populations. Chronic exposure to air pollution can induce inflammation and oxidative stress and impair immune function to increase infectious respiratory disease burden, particularly in vulnerable populations such as young children and older adults and individuals with pre-existing respiratory disorders. Excessive exposure to small particulate matter (PM_2.5_) pollution, for example, can magnify the adverse effect of climate change on respiratory health by altering the immune response and exacerbating the development of existing respiratory disease [28]. Chronic exposure to temperature and humidity changes can also influence innate and adaptive immunity by altering the regulation of specific immune responses. For example, extended exposure to elevated temperature can decrease the production of antigen-specific CD8^+^ T cells and antibodies [29]. However, excess disease risk associated with air pollutants and meteorological factors can be complex, as evident from a study that found that excess risk for TB was positively associated with NO_2_ level and windspeed; negatively correlated with O_3_ level, temperature, and relative humidity; and was not detectably associated with PM_2.5_ or SO_2_ concentration or sunshine duration [30].

Several steps should be taken at the individual, regional and global scale to prevent future EID outbreaks. First, surveillance of animal populations that could serve as potential EID reservoirs by effective surveillance system should be considered a key measure for the early recognition of pathogens that demonstrate potential for spillover events in human populations. Early identification of changes in such candidate EID reservoirs can permit interventions to prevent or attenuate an outbreak. These can include identification of candidate transmission routes and potential preventative measures, new pathogen targets for surveillance in at-risk regions, and for vaccine or drug development efforts. Second, substantial effort should be made to improve sanitation efforts in at-risk regions, particularly in areas most likely to have interactions with a pathogen reservoir that could lead to spillover or transmission events. This approach should focus on proper waste management and the safety of the water supply, particularly in low-income or underdeveloped regions. Third, significant effort should be applied to public education efforts that raise awareness of and provide information about the disease and its transmission routes in an accessible format, and suggest behavioural modification that can limit disease transmission (e.g. proper hand hygiene, mask usage, isolation of laboratory-confirmed patients, and quarantine of symptomatic individuals or those who were close contact of actively infected patients). Finally, since considering infectious diseases can readily spread across national borders, international organizations, governments and scientists should collaborate on approaches to detect EID pathogens in wastewater from major urban travel hubs (homes and hotels) and major sites of regional or international travel (bus/train stations and airports) to detect entry of these pathogens into new regions.

## Novel technologies for EID monitoring and screening

**Figure 4.**
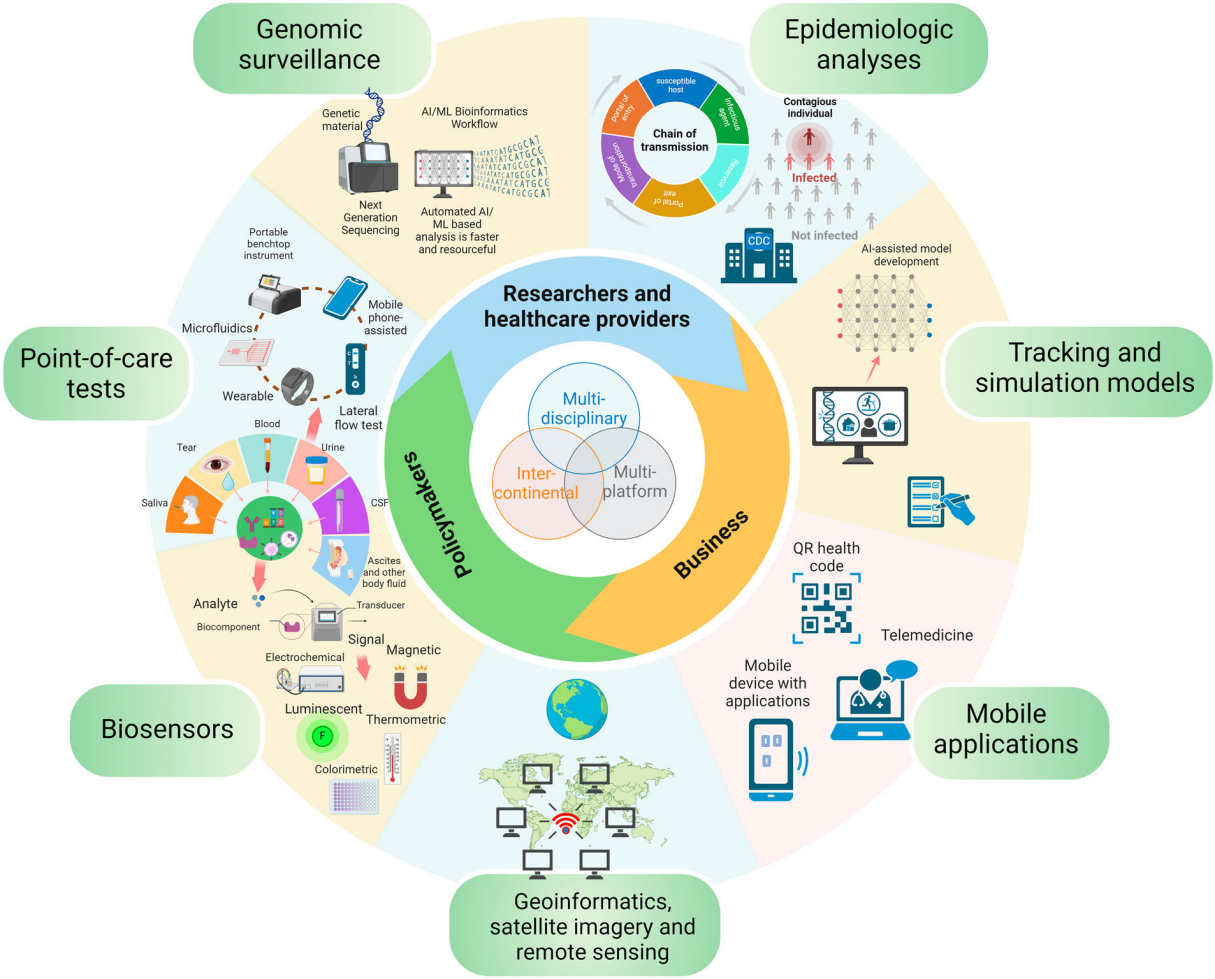
Multi-disciplinary, inter-continental and multi-platform-based approaches to prevent or contain EID, outbreaks, epidemics, and pandemics. Figure created with Biorender.com.

Sensitive new surveillance approaches that can detect new EID pathogens and diagnose new EID cases are needed to address the growing threat of climate-sensitive infections. Previous experience indicates that successful efforts to prevent or contain future EID outbreaks, epidemics, and pandemics require multi-disciplinary and inter-continental collaboration, and the integration of an array of innovative approaches on multi-platforms (Figure 4).

### Genomic surveillance

Lessons from efforts to control the COVID-19 pandemic highlight the significance of national and international genomic surveillance networks. The first outbreak of this new pathogen was detected as a series of pneumonia cases of unknown origin in Wuhan, China in December 2019. By January 2020 the responsible virus was identified as a novel coronavirus, SARS-CoV-2 [31], and its genome sequence had been released [32], and by July 2020 a dynamic phylogeny-informed nomenclature had been published to classify and track the growing genetic diversity of new SARS-CoV-2 lineages [33], which is reflected in the more than 16 million genomes shared through the Global Initiative on Sharing All Influenza Data (GISAID) as of November 2023 [34]. This process made it possible to detect and track the transmission of several SARS-CoV-2 variants of concern (VOCs), which demonstrated the potential for immune evasion or increased transmissibility, and highlighted the importance of genome sequencing and surveillance data in vaccine design and public health decisions [35].

Genomic surveillance data can, in conjunction with other data, provide insight into the source, development timeline, and transmission route(s) of an EID. For example, Zika virus genome sequence was obtained from infected patients and *Aedes aegypti* mosquitoes in the United States during a Zika outbreak in 2016 for epidemiologic tracking, which suggested the initial outbreak occurred several months before the first cases were detected in Florida in March 2016 [36]. This data also indicated that there were between four and forty distinct virus introduction events from the Caribbean that contributed to the outbreak, in agreement with the high Zika incidence rate and traffic between this region and Florida [36].

Genomic data can also provide vital information about emerging drug resistance in a pathogen of interest. For example, the rapid global dissemination of *Salmonella enterica* serovar Concord has raised significant concern as this serovar frequently exhibits multi-drug resistance. Genomic analysis of global isolates of this serovar obtained between 1944 and 2022 has revealed that it is a diverse polyphyletic serovar, and that four of its lineages found to primarily circulate within Ethiopia frequently exhibited multi-drug resistance due to anti-microbial genes that were transferred on structurally diverse IncHI2 and IncA/C2 plasmids or incorporated in their genomes [37]. Rapid lineage identification could thus provide a means to quickly assess the relative threat posed by new cases of this serovar and inform treatment decisions. Similarly, another genomic epidemiology study analyzed more than 2300 genomes of *S. enterica* isolates carrying mobile colistin resistance (*mcr*) genes that were collected over a 22-year period from diverse sources in 20 countries to analyze the global prevalence and transmission history and the ancestry and evolution rates of these strains [38]. This information is important since *mcr* genes associated with conjugative plasmid vectors, transposons, and insertion elements enable horizontal transfer of resistance to colistin, which is used to treat multi-drug resistant and extensively drug resistant infections. Further, this study focused on *S. enterica* sequence type 34 (ST34) lineage, which has displaced the “traditional” ST19 lineage, and was recently responsible for an outbreak affecting at least 11 countries. Genomic surveillance studies and networks can thus provide an important means to monitor the dissemination of key microbes that carry critical drug-resistance genes, particularly those capable of horizontal transmission.

Genomic surveillance data plays an essential role in guiding vaccine and drug development, production, and treatment strategies. However, genetic manipulation studies that target the genome of a host cell can also be employed to better understand the infection process and identify new therapeutic targets. For example, one group used a genome-scale CRISPR (clustered regularly interspaced short palindromic repeats)-based loss-of-function screen to identify genes whose deletion and pathways whose disruption conferred resistance to SARS-CoV-2 infection, and confirmed these findings using RNA interference and small molecular inhibitor directed at these targets [39].

Genomic surveillance data generated during an epidemic can enable rapid development of effective diagnostics and vaccines that account for new variants, and when combined with epidemiological information can provide a more detailed picture of ongoing transmission dynamics. Effective genomic surveillance efforts currently are limited primarily by lack of sequencing resources, current bioinformatic pipelines for data analysis, and regular sample collections workflows required to generate informative populations of specimens. Building effective surveillance networks can require collection of sequence data from representative populations to enable the sensitive detection of emerging or re-emerging pathogens with low prevalence during spillover events or early in an outbreak. Such genomic surveillance data should be accessible as a global health resource in both developed and underdeveloped areas, particularly during outbreaks, epidemics, and pandemics.

### Epidemiologic analyses

Epidemiologic data, ranging from aggregated case counts to contact tracing is required to characterize key parameters of an EID outbreak, including R_0_ and transmission heterogeneity of the pathogen, and the distributions of critical outcomes (e.g. time from symptom onset to hospitalization) [40]. Epidemiologic surveys are also often employed to understand the major routes of spread during an outbreak by performing close contact tracing studies. Such epidemiological investigations normally attempt to acquire contact and potential exposure histories, general demographic information, recent clinical symptom histories, and symptom onset and disease progression timelines. Ideally, this information can provide detailed timelines that can link the first and subsequently infected individuals through their close contacts and provide information about the characteristics required for infection. Such contract tracing efforts may also be valuable for endemic diseases. For example, a recent prospective epidemiological of TB-infected patients in rural China who were categorized into distinct genome-clusters using whole genome sequencing data from their culture specimens found that transmission mainly occurred via social contacts [41]. Thus, active screening and aggressive contact tracing efforts could benefit TB control in similar settings. Such investigations could also be aided by location data obtained from personal devices, although care would have to be taken to address privacy concerns. Similarly, it is tempting to integrate real-time oceanic and climate variability data with epidemiologic and dynamic demographic data of affected or at risk populations to improve the modelling of potential cholera outbreaks and design of cost-effective public health strategies [42]. Notably, GeoSentinel, a global surveillance and research network, has expanded to encompass 71 sites across 29 countries [43]. By using data from travellers and migrants who have traversed international borders, this network has successfully helped to identify multiple unrecognized outbreaks of public health importance (e.g. chikungunya from Myanmar 2019 [44] and Zika from Thailand 2022 [45]). Novel approaches that integrate epidemiologic and climate data with other data sources should enhance our understanding of and ability to predict the factors that lead to EID outbreaks and enhance disease transmission.

### Tracking and simulation models for EID transmission

Novel studies that integrate mathematical, biostatistical and computational methods, including artificial intelligence (AI), could permit integrated analysis of genomic data with corresponding epidemiologic, demographic, and climate or meteorologic data to improve the performance of predictive models. Extensive modelling efforts were employed during the COVID-19 pandemic to allow rapid estimates for regional outbreaks of SARS-CoV-2 and its variants to inform monitoring, coordination, and resource deployment efforts, using models that employed a variety of approaches, including data mining and AI/machine learning techniques [46,47]. For example, researchers used an adjusted negative binomial model to estimate the effectiveness of SARS-CoV-2 vaccine dosage in Hong Kong and predict that a third vaccine dose should be prioritized in aged and high-risk populations [48,49], in accordance with the call for optimizing dosing regimens particularly for special at-risk populations [48]. Models have also been developed to inform public health decisions for other EIDs caused by dengue, Zika, yellow fever, or West Nile viruses [50-53]. Results from these models can be applied to allocate health services and vaccine stocks, and inform vaccination strategies and public information campaigns.

EID policy decisions may require robust prediction models that accurately simulate transmission dynamics and infection cases to justify preventive measures and interventions that may generate controversy (e.g. social distancing and mask mandates, bans on large gatherings, and lockdown, quarantine, and confinement measures, and school closings). This may require the adoption of new state-of-the-art approaches that employ AI and/or machine learning and large integrated datasets to maximize prediction accuracy [47], as has been shown in the GeoSentinel Network [43].

### Geoinformatics, satellite imagery and remote sensing technologies

Geographic information system (GIS) can be used to collect and store geographically and temporally referenced information that can be used to visualize and analyze factors associated with EID outbreaks and develop models that reflect potential effects on EID outbreaks and disease transmission [54]. Satellite imagery and remote sensing approaches can, for example, identify interfaces between pathogen reservoirs and susceptible host species that may serve as hotspots for spillover events [55]. Satellite data could also potentially be used to provide early warning of an increased risk of an EID outbreak or potential spillover event by remotely monitoring parameters associated with these events (e.g. changes in vegetation coverage and river and lake areas, wind and dust storm frequency) to inform surveillance, vaccine planning, and health care decisions required identify and contain such outbreaks. This constitutes a fundamental advance from the basic mapping approaches historically used to track the spread of cholera, influenza, and other disease outbreaks to evaluate their transmission processes. New analytic methods can also simplify the analysis, visualization and detection of disease patterns, and more than a quarter of health-related GIS literature now focuses on mapping infectious diseases [56]. Geoinformatics and remote sensing can also play an important role in the study and control of epidemics by permitting geographic mapping, epidemiological modelling and location-based alert services [57]. These approaches have been applied to outbreaks of SARS, seasonal influenza [58] and, a highly pathogenic H5N1 avian influenza variant [55].

Satellites can also be employed in telemedicine programs to monitor health, diagnose, and evaluate treatment efficacy for multiple common conditions, including infections, by enabling real-time patient/physician visual interactions in populations that do not have direct or sufficient access to traditional health care sites [59]. Information from these interactions can also be used in epidemiology assessments, while employing satellites to collect data from wearable and remotes sensors can potentially be used to detect changes in human and animal populations to improve epidemiologic surveillance of disease transmission required to allow early interventions that can improve the emergency response to a disease outbreak [60]. This includes the potential for continuous real-time monitoring of multiple health parameters (e.g. heart and respiratory rates, blood pressure, body temperature, and blood oxygen level) that can provide valuable information about infection status and disease severity [61]. However, the value of the information obtained from geoinformatics, satellite imagery, and remote sensor data could be further enhanced by increased development of these resources through enhanced cooperation, investment in, and partnerships among space sector, public health, and humanitarian organizations [62].

### Mobile applications

EID surveillance systems should ideally incorporate multiple functions, which can include the collection of geoinformatics, satellite imagery, and remote sensor data for outbreak prediction studies; and the central reporting of test results to improve case reporting for epidemiologic and disease transmission analyses required to inform public health. Cell phone networks have been employed to build more coordinated infection surveillance systems due to the broad geographic network coverage that is required to serve cell phones used by most of the global population. Early dedicated mobile apps have been developed to facilitate efforts to improve leprosy screening [63], adherence to TB treatment [64] and HIV prophylaxis [65], and antibiotic resistance testing [66]. The number and variety of these apps have demonstrated marked growth recently, partly spurred by experience gained in response to the COVID-19 pandemic. One significant example is the integration of a quick-response (QR) health code into Alipay and WeChat, the two most widely used apps in China, which indicates the relative infection risk of the phone’s owner [67]. This score is based on a user’s recent clinic visit history, purchase of fever-reducing drugs, history of travel to regions with high COVID-19 case rates, close contact with confirmed or suspected COVID-19 cases, or self-reporting of COVID-19 symptoms, and can be used in epidemiological investigations, quarantine efforts, and other social control efforts. However, this approach utilizes substantial amounts of personal information and this could limit its adoption in other settings due to the fear of data disclosure and misuse. Integration of AI into phone apps can also improve disease surveillance, as demonstrated by a recent study that developed an app employing an image-based deep convolutional neural network to diagnose monkeypox virus infections from skin lesion images of individual with suspected infections without requiring any input from a healthcare professional [68]. Finally, while most apps exclusively focus on a single type of infection, a recent study developed a symptom-based surveillance app (ITIT: Infection Tracking in Travellers) designed to detect infections acquired during travel using real-time collected information to examine associations between travel and illness patterns [69]. Mobile apps thus have significant potential to improve surveillance and early diagnosis of EIDs. Future studies are expected to improve the sensitivity and accuracy of these apps, but effort should also focus on increasing the integration of collected data into disease surveillance networks while strengthening privacy protections measures for user information.

### Biosensors

To address the increased EID risk posed by climate change, there is a growing demand for new rapid, inexpensive, and user-friendly assays for various specimen types that can sensitively and accurately detect pathogens with significant potential to cause epidemics or pandemics. Substantial effort has been made to develop novel technologies and devices that fulfill these requirements. Biosensors, which combine a bioreceptor, a biological material capable of specific target recognition (e.g. a cell, antibody, enzyme, aptamer, or DNA/RNA probe), with a sensor (e.g. an electrochemical, optical, or mass-based transducer) that can detect target and bioreceptor interactions exhibit strong potential utility for such assays [70]. For example, one study has described the development of a biosensor that employs an antibody conjugated to a graphene field-effect transistor to rapidly detect SARS-CoV-2 in clinical specimens at a high sensitivity (limit-of-detection 2.4 × 10^2^ cfu/mL) [71].

Biosensors can be wearable, ingestible or implantable depending upon their specific purpose [72]. Wearable biosensors are frequently used to non-invasively monitor physiologic parameters that can provide evidence of infection (e.g. body temperature, heart rate, respiratory rate, blood oxygen saturation (SpO_2_), and blood pressure), but these biosensors can employ microneedles or other means to analyze biomarkers present in dermal interstitial fluid or other readily accessible body fluids. Ingestible biosensors can extensively interact with the gastrointestinal mucosa to analyze a range of biomarkers expressed by the mucosal immune system and gastrointestinal tissue that might otherwise be difficult to analyze without invasive specimen collection procedures. Implantable biosensors can be placed to directly contact a subcutaneous site or tissue of interest (e.g. brain tissue, circulatory system, etc.), allowing them to monitor important physiologic parameters and biomarkers *in situ* to allow early diagnosis and rapid monitoring of disease progression and response to treatment [72]. Implantable biomarkers are suitable for use in specific populations where other approaches are not effective or feasible. Wearable, ingestible, and implantable biosensor platforms have strong potential to improve disease detection, diagnosis, and monitoring in the general population, as biosensors can detect diseased-associated biomarkers in blood, urine, saliva, tear, interstitial fluid, and sweat with robust sensitivity and accuracy [73].

Next-generation diagnostic biosensor prototypes could, however, be further improved to enhance their potential adoption by advances in biomimetic and conformable materials, low-energy power harvesting/generation methods, wireless communication approaches, and signal transducer and detector sensitivity increases [74]. Advances in AI-based data analysis applications, bioinformatics, cloud-based data storage, the Internet of Things, and cellular communication in the biomedical sector could also enhance their utility and clinical adoption [75]. Notably, application of AI algorithms to the analysis of data from multiplex biosensors could significantly improve diagnostic accuracy to increase confident use of their results in clinical decisions.

### Point-of-care tests

Rapid and accurate POC tests could play a key role in attenuating infectious disease transmission by providing immediate diagnostic results at hospital and clinical sites. This would permit clinicians to make real-time diagnostic, treatment, and quarantine decisions while an individual is still available, avoiding the need and effort required to recontact them with their test results and potentially have them return for treatment, contact-tracing, and/or quarantine. Protein and serologic disease biomarkers specific for distinct viral and bacterial diseases, such as COVID-19, Lyme disease, and malaria, have been employed to develop rapid POC diagnostic tests [76]. However, advances in the development of bedside rapid molecular tests that rely on PCR, isothermal nucleic acid amplification, and/or CRISPR reactions have led to the rapid proliferation of health monitoring devices, and growth of direct-to-consumer and over-the-counter diagnostic testing capacity. New POC devices that employ nanotechnology, materials engineered with specific functions, creative signal detection methods, AI-based data analysis, and/or an internet-of-thing or other data communication approaches also exhibit strong potential to improve infectious disease control efforts [70]. For example, smart POC diagnostic devices that sync with a wireless system to securely communicate results to clinicians and regulatory agencies could improve patient data management, epidemiologic analyses, and the evaluation of vaccine and drug intervention studies [77]. This approach was taken by a lateral flow SARS-CoV2 immunoassay employing a 5G-enabled sensor that could communicate with an array of edge network devices (e.g. computers and cell phones) [78] to permit remote data reporting.

## Future perspectives

Climate change is expected to alter the incidence, prevalence, and distribution of EID outbreaks and to degrade host immunity in human and human-adjacent species, raising the potential for more frequent and severe epidemics by these pathogens. The SARS-CoV-2 pandemic, and the rapid global circulation of its variant strains, highlight the need for a collaborative, worldwide framework for infectious disease detection, prevention, and treatment that employs new technologies that have recently become available [79]. There is a growing need to forecast the future burden of specific EIDs under different climate change scenarios through models that integrate retrospective information from past epidemic or pandemics and climate conditions data [80]. It is vital to establish global strategies to track and model potential responses of candidate EIDs to altered climate conditions to project their future behaviour and guide research on early detection and diagnosis technologies and vaccine development for these targets. Multi-disciplinary collaborations will be required to develop effective inter-continental surveillance and modelling platforms that employ AI to mitigate climate change effects on EID outbreaks through interventions targeting all levels of this process, from the host reservoir onwards. Necessary changes will require coordinated responses and adequate funding supports from policymakers, businesses, and healthcare providers that may be needed to permit effective action from the community level to integrated regional, national, and international efforts.
